# Characteristics of Lethal Electrical Injuries in Central and Northeastern Bulgaria for a 27-Year Period (1980–2006)

**Published:** 2008-02-15

**Authors:** William Dokov

**Affiliations:** Department of Forensic Medicine and Deontology, Medical University of Varna, Varna, Bulgaria

## Abstract

**Objective:** Despite the advancement of forensic science, electro-traumas still pose serious challenges. **Methods:** We have studied the forensic medical documentation from 485 autopsies following electro-trauma over the period 1980–2006, performed at the forensic wards in 6 districts of the country The statistical analysis includes comparison of means and percentages. They are carried out using SPSS Version 11. We accepted statistical significant values of *P* equals; .05. **Results:** The incidence of lethal injuries caused by electricity is 1.29 cases per 100000 people per year. The average age of the deceased from electro-trauma is 37.3 years. Men (85%) prevails over women (14.84%). There are 24.32% of the cases that are work-related accidents, and 60.61% of them are domestic. Suicide through electrocution is relatively rare: 7.21%. Homicide has not been registered in our study. Low-voltage injuries (42.06%) are more common than high-voltage ones (30.72%). 62.68% of the lethal cases occur in summer, between June and September. **Conclusions:** Among the studied cases, electro-trauma occurs at a young age. The victims are typically men. Work-related accidents are more common than domestic ones; injuries by low voltage are observed more frequently than those by high voltage. Suicides are very rare, and not a single case of homicide has been observed in the study. There exists a seasonal variation in incidence of lethal accidents caused by electric current, its peak being during the summer months.

Despite the advancement of forensic science, electro-traumas (ET) still pose a seriouschallenge. The aim of the study is to examine the incidence and characterization of ET in some areas of Bulgaria as well as the victims' average age and their distribution by gender.

## METHODS

The study has been conducted in 6 districts of the Republic of Bulgaria on a total area of 16 910.31 km^2^ (15.23% ± 0.54% of the whole country's territory) and a population of 1 388 277 (17.98% ± 0.06% of the whole population).^1^ We have studied the forensic medical documentation from 485 autopsies caused by ET over the period 1980–2006, performed at the forensic wards in 6 districts of the country (Varna, Dobrich, Gabrovo, Ruse, Silistra, and Shumen). The statistical analysis includes comparison of means and percentages. They are carried out using SPSS Version 11. We accepted statistical significant values of *P* = .05.

## RESULTS

**Figure 1:**
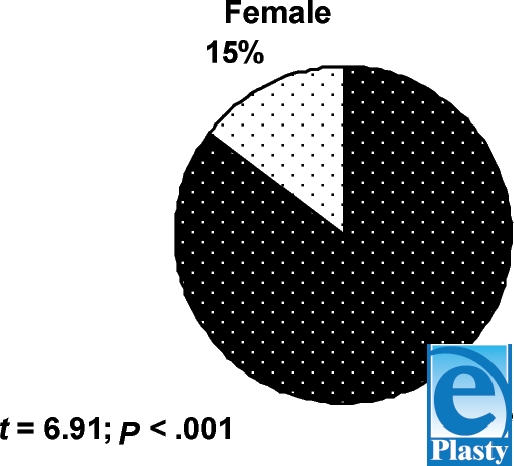
Electro-trauma victim distribution by gender.

For the 27-year-long period under study, 485 cases of ET have been autopsied, or 1.29 per 100000 people per year, with the population averaging 1 474 945 people^1^, ^2^ during the same period. The average age of the deceased because of ET is 37.3 ± 2.32 years (SD = 18.3). Men (85.15% ± 3.42%) (*n* = 413) prevail over women (14.84% ± 8.21%) (*n* = 72) (Fig 1) with a statistically reliable difference (*t* = 6.91; *P* < .001).

**Figure 2:**
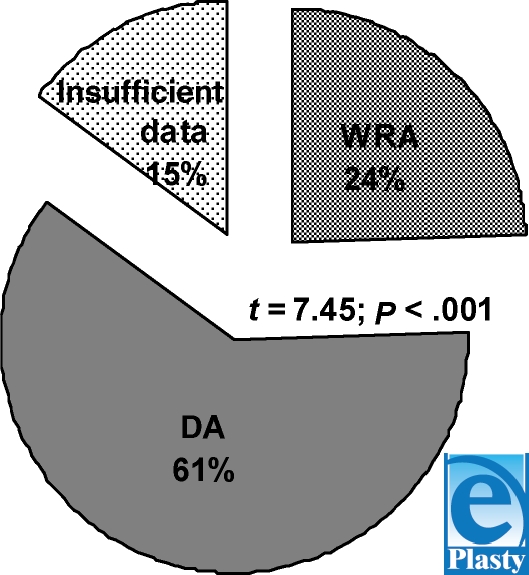
Electro-trauma victim distribution by cause of death. WRA, work related accidents; DA, domestic accidents.

24.32% ± 7.74% cases (*n* = 118) are work-related accidents (0.29 per 100000 people per year on average), and 60.61% ± 5.58% (*n* = 294) are domestic accidents (0.74 per 100000 population per year on average) (Fig 2). The difference is also statistically significant (*t* = 7.45; *P* < .001).

Suicides by electric current have rarely been observed 7.21% ± 8.56% (*n* = 35) or (0.09 per 100000 people per year on average). Homicides have not been registered in our article.

**Figure 3:**
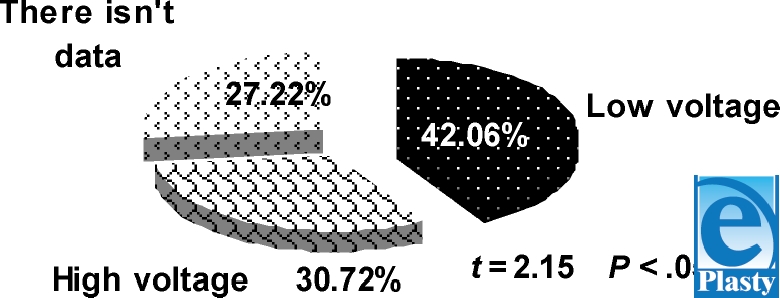
Electro-trauma victim distribution by type of voltage.

Injuries by low voltage (Fig 3) 42.06% ± 6.77% (*n* = 204) or (0.51 per 100000 people per year on average) are more common (*t* = 2.15; *P* < .05) than those by high voltage 30.72% ± 7.40% (*n* = 149), which is 0.37 per 100000 people per year on average.

**Figure 4:**
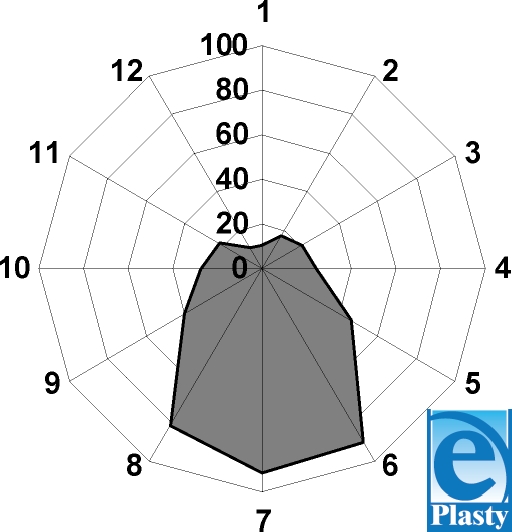
Electro-trauma victim distribution by calendar months.

Seasonality is clearly marked in our study (Fig 4). Thus, 304 cases are concentrated in the summer months from June to September, which is 62.68% ± 5.43% of all deceased from electric current.

**Figure 5:**
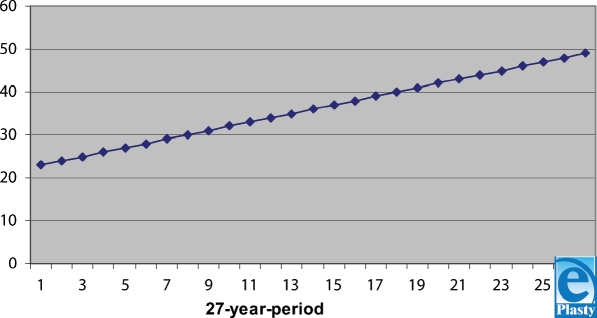
Ascending linear trend of the electricity-caused injuries in the studied area.

Fewer cases are observed in December: 10 (2.06% ± 8.8%), and the most in July: 92 (18.96% ± 8.00%), with significant difference (*t* = 2.8; *P* < .001). We used the smallest-square method to draw a trend of the electricity-caused injuries in the studied area. The resultant graph (Fig 5) clearly shows an ascending trend.

## DISCUSSION

The analysis of our results shows that the registered average age of the deceased from ET is higher than that in other studies,^3^,^4^ and that male gender is predominantly affected—a fact observed not only by us^5^ but also by other authors.^3^,^4^

Suicides by electric current are relatively rare: 7.21%.^6^ Homicides have not been registered in our study, which implies that they are extremely rare.^7^.

We have found that deaths following low voltage injuries are prevalent. In the literature, there is not a unanimous opinion in this respect^8^,^9^. We consider that the larger number of lethalaccidents caused by low-voltage electricity result from the immensely larger access to electrical installations and low-voltage operated electrical devices.

Similar to the studies conducted by others authors, 4 we have ascertained seasonality in fatal accidents caused by electric current whose number increases abruptly during the summer months.

We attribute this seasonal trend to the following major factors. The electrical threshold of the myocardium for excitability is decreased with increased environmental temperature and hence the increased number of cases of ventricular fibrillations. When environmental temperature is high, skin moisture is increased and skin resistance is decreased. The summer season presupposes less clothing, and a greater lilihood of direct electrical contact with moist bare skin.

## CONCLUSIONS

Among the studied cases, ET occurs in young age groups. The victims are typically men. Work-related accidents are more common than domestic ones, injuries by low voltage are observed more frequently than those by high voltage. Suicides are very rare, and not a single case of homicide has been observed in our study. The incidence of lethal accidents vary with season, increasing during the summer months.
